# Statistics in Trauma Research

**DOI:** 10.5812/traumamon.17606

**Published:** 2014-03-18

**Authors:** Hamidreza Rasouli, Maryam Rezaee, Zahra Danial

**Affiliations:** 1 Trauma Research Center, Baqiyatallah University of Medical Sciences, Tehran, IR Iran

**Keywords:** Wounds and Injuries, Statistics, Designs

*Dear Editor,*

Statistics is a science with many applications in trauma research and several other fields. Prior to conducting research, the study problem must be clearly defined; then, measurement tools for the study variables should be determined, next, data collection instruments as well as survey questionnaires should be obtained (1). Selection of the study design and sampling method depends on the available equipment and aim of the study. Generally, there are two types of studies:

1. Observational studies and
2. Experimental studies (clinical trial).

In an observational study, researchers observe cases and measure the variables of the study without any interventions in the cases (2). In an experimental study, the researcher determines who is treated (2). Cross-sectional, case-control, and prospective (or cohort) are three different study designs of observational studies. In a clinical trial, the researcher randomly allocates participants to groups; while trying to keep all factors constant or controlled for comparing the outcomes between the groups (minimizing confounders). Prospective studies such as clinical trials provide better clues of causality than retrospective studies, but are often more difficult and sometimes impossible to perform. To find answers of study questions in invasive procedures, randomized controlled trials are not ethical to perform. In addition, in some situations, it is not ethical to divide participants randomly in a trial where one intervention option is surgical. Considering the restrictions in applying randomized clinical trials in surgical studies, observational studies are common to investigate the association between variables. Therefore, appropriate observational studies are very important and can provide true results.

In the next step, we should perform the appropriate data analysis and statistical modeling. In simple situations, computing indexes such as confidence interval, P value, standard deviation, standard error, or applying statistical tests may be sufficient. In many applications, the rule is to disprove the null hypothesis if they obtained P value is less than 0.05. Parametric methods include paired t-test, t-test and analysis of variance (ANOVA); nonparametric methods include chi-squared test, Wilcoxon signed-rank test, Mann-Whitney test, Kruskal-Wallis test, and the log-rank test. 

Correlation is a common matter when the aim is to evaluate the relationships between variables.

For quantitative variables, depending on normal or non-normal data distribution, parametric or nonparametric methods should be used, respectively. For situations in which the data is a sample from a normal or near normal distribution, parametric methods are generally more efficient than non-parametric methods. Two common tests to check the normality of data are the Kolmogorov-Smirnov test and the Shapiro-Wilk test. Depending on the normality of data, Pearson or Spearman correlation should be used. The most sophisticated study in traumatic injury research needs advanced statistical approaches, including modeling as well as testing of statistical models, to suitably interpret the data. Topics familiar to professional statisticians such as logistic regression, called generalized linear models are playing an increasing role in clinical studies. Logistic regression is suitable when only two possible outcomes are expected. Medical studies require a statistical expert consultation or a medical scientist, specifically educated in statistics.
